# Youth exposure to unhealthy digital food marketing in relation to race/ethnicity and income adequacy in Canada

**DOI:** 10.1186/s40795-025-01148-5

**Published:** 2025-08-11

**Authors:** Laura Vergeer, Carolina Soto, Mariangela Bagnato, Elise Pauzé, Ashley Amson, Tim Ramsay, Dana Lee Olstad, Vivian Welch, Monique Potvin Kent

**Affiliations:** 1 School of Epidemiology and Public Health, Faculty of Medicine, University of Ottawa, Ottawa, ON Canada; 2 Interdisciplinary School of Health Sciences, University of Ottawa, Ottawa, ON Canada; 3 Ottawa Hospital Research Institute, Ottawa Hospital, Ottawa, ON Canada; 4 Department of Community Health Sciences, Cumming School of Medicine, University of Calgary, Calgary, AB Canada; 5 Bruyère Research Institute, Ottawa, ON Canada

**Keywords:** Food marketing, Digital media, Youth, Race/ethnicity, Income

## Abstract

**Background:**

Youth of racial/ethnic minority groups and lower-income households are disproportionately exposed to unhealthy food marketing on television; however, there is limited evidence concerning digital marketing. This study examined differences in Canadian youth’s exposure to digital food marketing by race/ethnicity and household income adequacy.

**Methods:**

Frequency of food marketing exposure via digital platforms and digital food marketing techniques were self-reported by 996 youth in Canada aged 10–17 years. Proportional odds and logistic regression models explored differences between racial/ethnic (White vs. racial/ethnic minority) and income adequacy groups (low vs. medium vs. high), adjusted for sociodemographic and digital device usage variables.

**Results:**

White participants had lower odds of more frequent exposure to digital marketing of sugary drinks (OR: 0.70; 95% CI: 0.52–0.94), sugary cereals (OR: 0.56; 95% CI: 0.42–0.76), fruits/vegetables (OR: 0.63; 95% CI: 0.45–0.87), salty/savoury snacks (OR: 0.63; 95% CI: 0.47–0.85), fast food (OR: 0.74; 95% CI: 0.55–0.99), and desserts/sweets (OR: 0.68; 95% CI: 0.50–0.91) than racial/ethnic minority youth. Compared to youth from low income adequacy households, those with medium income adequacy were less likely to report more frequent exposure to marketing of sugary drinks (OR: 0.67; 95 CI: 0.51–0.89), fast food (OR: 0.66; 95% CI: 0.50–0.87), and desserts/sweets (OR: 0.65; 95% CI: 0.49–0.87). White youth were less likely than racial/ethnic minority youth to report exposure to unhealthy food marketing on ≥ 1 social media platform(s) (OR: 0.45; 95% CI: 0.30–0.68) and gaming/TV/music streaming platform/website(s) (OR: 0.71; 95% CI: 0.51–0.99); no differences were observed between income groups. White youth were less likely than racial/ethnic minority youth to report exposure to marketing featuring incentives/premiums (OR: 0.72; 95% CI: 0.52–0.99) and cross-promotions (OR: 0.71; 95% CI: 0.51–0.99). Participants of higher (OR: 0.68; 95% CI: 0.49–0.95) and medium (OR: 0.69; 95% CI: 0.50–0.93) income adequacy were less likely to report exposure to marketing featuring celebrities than those with low income adequacy.

**Conclusions:**

Youth of racial/ethnic minorities report more frequent exposure to digital food marketing, especially for unhealthy foods, than White youth in Canada. Differences were also observed between income groups. Comprehensive marketing regulations are needed to limit all youths’ exposure to unhealthy digital food marketing.

**Supplementary Information:**

The online version contains supplementary material available at 10.1186/s40795-025-01148-5.

## Background

Diets of lower nutritional quality are associated with an elevated risk of obesity and other non-communicable diseases (e.g., cardiovascular disease, cancer, type 2 diabetes) [1]. The diets of children in Canada are typically characterized by high intakes of ultra-processed, energy-dense foods and beverages (hereinafter referred to as “foods”) that are higher in sodium, free sugars and saturated fats, and low intakes of whole foods recommended by national dietary guidelines (e.g., fruits and vegetables, plant proteins) [2–5]. Poor quality diets are particularly prevalent among children and adolescents (hereinafter referred to as “youth”) of racial/ethnic minority groups and lower-income households in Canada and the US [6–11]. People of racial/ethnic minority and socioeconomically disadvantaged groups are also disproportionately impacted by diet-related diseases [12].

Marketing of unhealthy foods to youth is a primary contributor to poor diet quality. Youth are cognitively vulnerable and easily influenced by marketing [13]. As such, frequent exposure to powerful (i.e., persuasive) marketing of unhealthy foods can shape their preferences and food choices, leading to high intakes of these products that can persist into adulthood [14, 15]. Youth exposed to unhealthy food marketing are more likely to consume energy-dense products higher in sugar, sodium and saturated fat, elevating their risk of obesity and other diet-related chronic diseases [14]. Digital food marketing to children has become increasingly prevalent and concerning in recent years, particularly as children are spending more time online and are being introduced to digital media at younger ages [16]. A study of Canadian parents reported that 36% of children aged 10–13 years old spend ≥ 3 h online for leisure purposes per day [17]. Furthermore, approximately 50% of children (aged 7–11 years) and 87% of adolescents (aged 12–17 years) in Canada have their own mobile device [18]. The growth in advertising spending on digital food marketing is also concerning, with an estimated $74.1 million CAD spent on food advertising via digital media in Canada in 2019 [19]. Evidence suggests that youth of racial/ethnic minority groups and lower household incomes tend to spend more time online than those identifying as White and with higher incomes [20, 21]. At the same time, youth from lower-income households typically own fewer personal electronic devices and have less access to family devices than children of higher-income households [21].

Digital marketing uses highly engaging and targeted techniques to promote content that users are encouraged to share within their social networks, thereby increasing marketing power and impact [15, 22, 23]. Studies have demonstrated that youth in Canada are exposed to high volumes of marketing on websites popular with their age group, and on websites owned by food companies [24–30]. However, less is known about youth’s exposure to unhealthy food marketing via newer, more novel forms of digital media, such as social media websites and apps, TV streaming services, and video game livestreaming platforms, among others. While there is some evidence of Canadian youth being frequently exposed to unhealthy food marketing on social media [25, 27], these studies did not differentiate between platforms or examine differences between racial/ethnic or socioeconomic groups.

Children of racial/ethnic minority groups and socioeconomically disadvantaged backgrounds are often more frequently exposed to unhealthy food advertising and tend to be more receptive to marketing messages [31]. Studies from the US have shown that Black youth are exposed to a significantly higher frequency of food advertisements on television than their White peers [31–36]. Moreover, exposure to unhealthy food advertisements on television is greater among American youth from lower-income households, compared to those from higher-income households [31, 33, 35]. There is less evidence concerning socioeconomic and racial/ethnic differences in children’s exposure to unhealthy food ads in settings other than television [31, 37], such as digital media, and there has been very little research on this topic in Canada [24]. This may be, at least in part, related to the relative recency of the shift from traditional (e.g., broadcast television) to digital media (e.g., streaming services), and the rapidly growing and evolving nature of digital media platforms and marketing techniques. One survey examining self-reported exposure to digital food marketing in Canadian youth found that those of racial/ethnic minority groups and households with lower income adequacy reported more frequent exposure to unhealthy food marketing, compared with White and higher-income participants, respectively [38]. While this study examined exposures across multiple media and settings (e.g., TV, online, retail, print, school), there was limited examination of digital media, with no disaggregation by digital media platforms or marketing techniques. Newer online platforms (e.g., television streaming services) were also outside of the scope of the survey. This is, however, an important knowledge gap in Canada, given the increasingly diverse forms of digital media to which youth have access, subsequently increasing their time online [39, 40].

Implementing policies aimed at protecting youth from the harmful effects of food marketing could help to reduce dietary and health inequities [15]. In Canada, food marketing to children is largely self-regulated by the food industry through a voluntary commitment to only market foods to children that meet their nutritional criteria, known as the Code for the Responsible Advertising of Food and Beverage Products to Children (CCFBA, preceded by the Canadian Children’s Food and Beverage Advertising Initiative, CAI). The CCFBA was developed by the Association of Canadian Advertisers, the Canadian Beverage Association, Food, Health & Consumer Products of Canada and Restaurants Canada, and is administered by Ad Standards, the Canadian advertising industry self-regulatory body [41]. However, studies from Canada and elsewhere have consistently shown that self-regulatory policies often leave children exposed to frequent and powerful marketing of unhealthy foods [15, 42]. Quebec is currently the only province with mandatory restrictions that apply to food advertising to children. Its Consumer Protection Act bans all commercial advertising directed at children under 13 years of age in various media, including digital media and most settings [43]. At the federal level, regulations to limit unhealthy food marketing to children were first proposed in 2016, but have yet to be passed or implemented, and the most recently proposed restrictions would only apply to children under 13 years of age [44].

Despite evidence from other countries (primarily the US, but also the UK and Australia) to suggest that children of racial/ethnic minority groups and of households with lower incomes are more exposed and vulnerable to unhealthy food marketing [31], there is a need to further explore this relationship in Canada, a multicultural country where more than 30% of the population identifies as a racial/ethnic minority group and approximately 10% and 22.9% are considered low-income and food insecure, respectively [45–47]. Such research is particularly needed on digital media, where youth spend a significant proportion of their time [16]. The purpose of this study was therefore to examine how youth’s self-reported exposure to digital food marketing in Canada differs by race/ethnicity and household income adequacy in terms of frequency and digital setting of exposure (e.g., TikTok, YouTube, Netflix) and digital marketing techniques. It was hypothesized that youth identifying as racial/ethnic minority groups and those from households of low income adequacy would report more frequent exposure to digital marketing of unhealthy foods than White youth and those from medium or high income adequacy households.

## Methods

### Study protocol

The data for this study were derived from an observational cross-sectional survey administered in April 2023, focusing on youth aged 10 to 17 years residing in Ontario or Quebec, Canada’s two most populous provinces with very different policy environments concerning food marketing to children. The purpose of the survey was to investigate youth’s self-reported exposure to digital food marketing (e.g., on social media, websites, streaming platforms). The survey was developed using items from the Canadian census, the International Food Policy Study Youth Survey [48], previous research on digital food marketing [49, 50], and a preexisting survey about media use developed by our group and administered to youth in Canada [27]. The surveys administered to parent and youth participants of this study are provided in Supplementary Tables 1 and 2. Leger Marketing [51] and their affiliated partner Quest Mindshare [52] recruited participants stratified by sex (600 males, 600 females), province (Ontario, Quebec) and age (10–12 years, 13–17 years).

Parent panelists were invited by email to take part in the research. Eligibility criteria required them to be residents of Ontario or Quebec and have a child aged 10–17 years who had access to a digital device capable of completing an online survey. The survey was available in both English and French. Parent participants responded to a brief survey to collect sociodemographic data about their child and household. Participating youth were subsequently surveyed to gather data about their exposure to unhealthy food marketing across a variety of digital media platforms in the 7 days preceding the survey. Compensation for participants who took the survey included either cash or virtual incentives that could be redeemed for gift cards. Ethics approval was received from the University of Ottawa Research Ethics Board (file H-11-21-7343). In terms of quality control, filter questions were used to flag participants who completed the survey too quickly and/or inattentively, open-ended responses were checked for nonsensical or gibberish responses, and respondents who finished the survey in under one-third of the median completion time were flagged; data for these participants were excluded. Parent/guardian and youth participants signed consent forms before completing the survey. The study protocol is also described in previously published studies based on this survey data [53, 54].

### Measures

#### Self-reported exposure to digital food marketing

The survey asked youth to indicate their frequency of exposure to marketing of sugary drinks (e.g., soda/pop, sports drinks, energy drinks, juices), sugary cereals, fruits or vegetables, salty/savoury snacks (e.g., potato chips, pretzels, cheese puffs), fast foods (e.g., pizza, French fries, burgers), and desserts or treats (e.g., cookies, ice cream, candy) on their smartphone, tablet or laptop/desktop computer in the 7 days preceding the survey. For each type of advertisement (e.g., sugary drinks), participants could select one of the following response options: “never”; “1–3 times during the week”; “4–6 times during the week”; “every day”; “more than once a day” and “prefer not to answer”.

Youth also reported if they recalled seeing or hearing advertisements for ‘unhealthy’ food or drinks (e.g., packaged foods high in sugar, salt or fats, such as soda/pop, fast food, chips, sugary cereals, cookies, and chocolate bars) in the past 7 days on any of the following social media platforms (answering separately for each platform): Facebook; Instagram; X (formerly known as Twitter); TikTok; Snapchat; YouTube; Pinterest; blogs or websites; posts and videos shared by influencers; or posts and videos shared by friends on social media. Similarly, participants indicated whether they had been exposed to marketing of unhealthy foods on gaming, TV or music streaming platforms/websites (answering separately for each): Twitch; gaming websites (e.g., Roblox); livestreamed games or eSports; TV streaming platforms (e.g., Disney+, Netflix); or Spotify.

Finally, participants were asked to indicate which digital marketing techniques they had seen used in advertisements of unhealthy food or drinks in the past 7 days. Participants were asked about their exposure to each of the 25 marketing techniques examined in this study, which were subsequently categorized as follows (based on Health Canada’s categorization of marketing techniques used in their 2023 policy proposal for restrictions on food marketing to children [44]): (1) characters or child/teenage actors (including cartoon characters from movies or TV, characters owned by food companies, and child or teenage actors); (2) child-appealing subjects, themes and language (including slang used by children or teenagers, themes like magic, mystery, adventure or heroes, as well as themes like fun, popularity, being cool, being fashionable, being free and independent); (3) celebrities, public figures and sports (including celebrities from movies/TV/bands, social media influencers, athletes or favourite sports teams, and extreme sports); (4) child-appealing visual design, audio and special effects (including appealing bright colours, catchy/popular music, and special effects/animation); (5) references to product benefits, health or nutrition (including highlighting new products, product convenience, and referencing health or nutrition); (6) incentives and premiums (including contests, free giveaways, limited time offers, price promotions, and reward/incentive programs); (7) cross-promotions (including a link to an event, movie or TV show); and (8) engagement techniques (including activities/polls/games/quizzes, and encouragement to like, comment or share content with friends on social media).

#### Use of digital devices

Youth indicated if they owned a smartphone, a tablet and/or a laptop or desktop computer, or if they used the devices of family members. Based on these data, it was determined whether participants reporting owning no devices or at least one, two or three types of devices. Participants also indicated how much time they typically spend on various online activities (on both weekdays and weekend days), including: watching YouTube; on social media (including messaging, posting, or liking posts on platforms such as Instagram, X (formerly Twitter), TikTok, Snapchat, Facebook); watching TV shows or movies on television streaming platforms (e.g., Disney+, Netflix); playing games on smartphones, computers or game consoles; browsing online, reading websites, Googling; and watching gaming or livestreaming content on Twitch. Estimates of total screen time on weekdays and weekend days were calculated using the sum of exposure (in minutes) across all types of digital media. Using a non-parametric Winsorization approach, digital screen times in the top 5% (i.e., extreme values) were lowered to the 95th percentiles (weekdays = 1080 min; weekend days = 1140 min). A weighted total screen time was then estimated by the sum of the Winsorized weekday (weighted 5/7) and weekend day (weighted 2/7) screen times.

#### Sociodemographic characteristics

Parents were asked to provide data on their family’s province of residence (Ontario or Quebec) and household income adequacy, as well as their child’s age, sex, race/ethnicity, and height and weight. Age was collapsed into two categories (10–12 years, 13–17 years) to align with the fact that marketing policies (including the Consumer Protection Act, and CCFBA, and federal restrictions being considered [41, 43, 44, 55, 56]) apply to children under 13 years. Parents were asked to select the racial categories that best described their child from the following options: Black (e.g., African, Afro-Caribbean, African Canadian); East Asian (e.g., Chinese, Korean, Japanese, Taiwanese descent); South Asian (e.g., Indian, Pakistani, Bangladeshi, Sri Lankan, Indo-Caribbean); Southeast Asian (e.g., Cambodian, Filipino, Indonesian, Thai, Vietnamese, or other Southeast Asian descent); Indigenous (e.g., First Nations, Métis, Inuk/Inuit); Latin American (e.g., Latin American, Hispanic); Middle Eastern (e.g., Arab, Persian, West Asian descent including Afghan, Egyptian, Iranian, Lebanese, Turkish, Kurdish); White (e.g., European descent); other race category; and ‘prefer not to answer’. Participants indicating multiple races were grouped into an ‘other or mixed’ race/ethnicity group category. Due to the limited number of observations in some racial/ethnic minority group categories, responses were combined into two broader categories: “White” and “racial/ethnic minority”, where the latter encompassed all responses except “White” and “prefer not to answer”. Participants’ BMIs were determined using their heights and weights as provided by their parents. The World Health Organization’s (WHO) body mass index (BMI) for-age cut-offs for youth were used to classify participants as having “severe thinness”, “thinness”, “normal weight”, “overweight” or “obesity” [57]. Extreme BMI values were identified by Z-scores greater than 4 or less than − 4. Responses of ‘severe thinness’ and ‘thinness’ were collapsed into one category due to few observations. Lastly, income adequacy was assessed by asking parents how difficult or easy it was to make ends meet based on their total monthly income, with the response options: “very difficult”, “difficult”, “neither easy nor difficult”, “easy”, “very easy”, or “prefer not to answer” [58]. This variable was collapsed into “low” (“very difficult” or “difficult”), “medium” (“neither easy nor difficult”) and “high” (“easy” or “very easy”) levels of income adequacy. A breakdown of racial/ethnic category and income adequacy category responses prior to the groupings is provided in Supplementary Table 3.

### Statistical analysis

The study sample excluded participants who responded “prefer not to answer” for the questions about digital food marketing exposure, race/ethnicity, income adequacy or any other sociodemographic or digital device ownership/usage variables (*n* = 215). Because the heights and weights or BMI values for many participants were missing or extreme (*n* = 266), an “extreme values/missing” group was formed and these participants were retained in the sample, similar to prior studies [59–65]. The final analytic sample included 996 participants.

Descriptive statistics were calculated for the sociodemographic characteristics, digital device ownership and usage variables and reported by race/ethnicity and income adequacy group and for the entire sample. The proportion of respondents who reported each frequency of exposure to digital marketing of the various types of foods was also calculated for each race/ethnicity and income adequacy group. For the digital setting of unhealthy food marketing exposure variables, proportions are presented for each individual digital platform (e.g., Facebook, Twitch), and for the two overarching digital platform categories: (1) social media; and (2) gaming/TV/music streaming platforms/websites (where the proportion reflects exposure to at least one platform within that category). Similarly, the proportion of participants who reported exposure to each marketing technique is reported, as is the proportion of participants who were exposed to one or more techniques within each category (e.g., celebrities and public figures, engagement techniques).

Individual proportional odds logistic regression models were used to examine associations of race/ethnicity and income adequacy group with self-reported frequency of exposure to each type of digital food marketing (sugary drinks, sugary cereals, salty or savoury snacks, fruits and vegetables, fast food, or desserts and sweet treats). Responses of “every day” and “more than once a day” were merged for these analyses because of small cell counts. Race/ethnicity and income adequacy (as categorical variables) were the independent variables of interest and models included province, age group, sex, BMI classification, digital device ownership and time spent on digital media as covariates. The latter two variables were adjusted for because differences exist between sociodemographic groups in terms of digital device ownership/usage and time spent on digital media [20, 66], and digital marketing is personalized and targeted based on online behaviours and user profiles [67]. These variables may therefore influence the frequency and power of digital food marketing to which youth with at least one digital device are exposed. Research also suggests a positive association between screen time and exposure to food marketing [59]. BMI was included as a covariate given the evidence of relationships of BMI/obesity with food marketing exposure and media consumption patterns and behaviours, and with race/ethnicity and income in youth [20, 68–71]. Binary logistic regression models explored differences between race/ethnicity and income adequacy groups in their exposure to unhealthy food marketing on ≥ 1 social media platform(s) and on ≥ 1 gaming/TV/music streaming platform(s)/website(s), adjusting for the same covariates as in the proportional odds models. Binary logistic regression models also examined differences between race/ethnicity and income adequacy groups in exposure to each category of digital food marketing techniques, with adjustment for the same covariates as in previous models. Lastly, proportional odds models investigated differences in the number of different marketing techniques that participants were exposed to (ranging from 0 to 25), while adjusting for the same variables as previous models. Based on consultation with a statistician (T.R.), the proportional odds assumption was not formally tested for all models, given we only sought to examine the significance and direction of associations (not treatment effects) and the proportional odds model is a generalization of the non-parametric Wilcoxon Mann-Whitney and Kruskal-Wallis tests [72]. IBM SPSS Statistics (Version 29.0.1.0) was used for the analyses and statistical significance was defined as *p* < 0.05.

## Results

### Sample characteristics

Overall, participants were approximately evenly distributed between age groups (50.2% aged 10–12 years), sexes (51.1% male) and provinces (50.0% from Ontario; Table 1). For the age group and sex variables, proportions were approximately equal across race/ethnicity and income adequacy groups, and between provinces for the income adequacy groups. However, of participants who identified as a racial/ethnic minority group, the majority were from Ontario (81.9%), with only 18.1% of participants from Quebec identifying as a racial/ethnic minority group. Additionally, most of the sample reported owning at least one type of digital device (computer and/or tablet and/or smartphone), with 39.9% owning at least two types of devices and some differences between race/ethnicity and income adequacy groups. For example, of those who identified as White, 29.0% reported owning at least one type of digital device, compared with only 19.8% of participants of racial/ethnic minority groups. The mean amount of time spent online per day was 435.5 min (SD: 264.6 min) for the total sample. Mean (±SD) time spent online per day was comparable between racial/ethnic groups (White: 434.5±263.0 min; racial/ethnic minority group: 438.6±269.9 min). Participants from households with low income adequacy reported spending more time online per day, on average (480.6±280.1 min), compared with those of high (416.4±254.7 min) or medium income adequacy households (409.2±252.5 min). Differences between racial/ethnic and income adequacy groups were also observed for specific digital platforms and online activities. For example, White participants reported spending more time on TV streaming platforms (88.1±81.3 min) and playing games (88.1±88.4 min) on weekdays, while participants of other races/ethnicities spent more time browsing/reading websites/Googling (58.6±64.8 min) or livestreaming on Twitch (36.9±64.6 min *)*. Additionally, participants of lower income adequacy households reported spending considerably more time on social media on weekdays (97.1±93.4 min) and weekend days (114.0±101.8 min) than their peers from medium (weekdays: 76.6±86.9 min; weekend days: 93.7±95.1 min) or higher income adequacy groups (weekdays: 78.2±84.7 min; weekend days: 89.5±86.8 min).

**Table 1:** Sociodemographic characteristics and digital device ownership and use among all participants (*n* = 996)

	Race/ethnicity		Income adequacy			Total sample (*n* = 996)
	White (*n* = 753)	Racial/ethnic minority^a^ (*n* = 243)	Low (*n* = 338)	Medium (*n* = 373)	High (*n* = 285)	
	*n* (%)	*n* (%)	*n* (%)	*n* (%)	*n* (%)	*n* (%)
Age (years)						
10–12	390 (51.8)	110 (45.3)	169 (50.0)	197 (52.8)	134 (47.0)	500 (50.2)
13–17	363 (48.2)	133 (54.7)	169 (50.0)	176 (47.2)	151 (53.0)	496 (49.8)
Sex						
Male	392 (52.1)	117 (48.1)	170 (50.3)	187 (50.1)	152 (53.3)	509 (51.1)
Female	361 (47.9)	126 (51.9)	168 (49.7)	186 (49.9)	133 (46.7)	487 (48.9)
Province						
Ontario	299 (39.7)	199 (81.9)	191 (56.5)	182 (48.8)	125 (43.9)	498 (50.0)
Quebec	454 (60.3)	44 (18.1)	147 (43.5)	191 (51.2)	160 (56.1)	498 (50.0)
BMI						
Severe thinness or thinness	24 (3.2)	8 (3.3)	12 (3.6)	12 (3.2)	8 (2.8)	32 (3.2)
Normal weight	371 (49.3)	97 (39.9)	141 (41.7)	181 (48.5)	146 (51.2)	468 (47.0)
Overweight	129 (17.1)	23 (9.5)	58 (17.2)	46 (12.3)	48 (16.8)	152 (15.3)
Obesity	61 (8.1)	17 (7.0)	45 (13.3)	21 (5.6)	12 (4.2)	78 (7.8)
Extreme/not stated	168 (22.3)	98 (40.3)	82 (24.3)	113 (30.3)	71 (24.9)	266 (26.7)
Computer (laptop or desktop), tablet and smartphone ownership						
Owns all three types of devices	175 (23.2)	82 (33.7)	96 (28.4)	93 (24.9)	68 (23.9)	257 (25.8)
Owns at least two types of devices	297 (39.4)	100 (41.2)	134 (39.6)	148 (39.7)	115 (40.4)	397 (39.9)
Owns at least one type of device	218 (29.0)	48 (19.8)	83 (24.6)	105 (18.2)	78 (27.4)	266 (26.7)
Does not own a device	63 (8.4)	13 (5.3)	25 (7.4)	27 (7.2)	24 (8.4)	76 (7.6)
Mean (SD)						
Self-reported time spent online per day, weighted (minutes)^b^	434.5 (263.0)	438.6 (269.9)	480.6 (280.1)	409.2 (252.5)	416.4 (254.7)	435.5 (264.6)
Weekday: Self-reported time spent online per day (minutes)						
Watching YouTube	90.9 (81.2)	89.7 (79.3)	98.9 (85.0)	87.1 (80.9)	85.3 (74.4)	90.6 (80.7)
On social media	84.5 (90.4)	82.4 (84.6)	97.1 (93.4)	76.6 (86.9)	78.2 (84.7)	84.0 (89.0)
Watching TV streaming platforms	88.1 (81.3)	79.0 (70.3)	91.7 (79.6)	86.1 (80.4)	78.7 (75.3)	85.9 (78.8)
Playing games	88.1 (88.4)	76.5 (78.0)	94.4 (90.9)	77.9 (81.8)	84.0 (84.9)	85.2 (86.1)
Browsing online, reading websites, Googling	42.1 (51.2)	58.6 (64.8)	50.3 (60.6)	40.7 (47.4)	48.3 (57.6)	46.1 (55.2)
Watching gaming or livestreaming on Twitch	21.0 (47.2)	36.9 (64.6)	31.6 (59.3)	19.8 (46.4)	23.5 (50.4)	24.9 (52.4)
Weekend day: Self-reported time spent online per day (minutes)						
Watching YouTube	113.5 (85.2)	115.9 (84.6)	125.4 (90.0)	111.4 (84.5)	104.1 (78.0)	114.1 (85.0)
On social media	100.0 (97.1)	97.5 (91.1)	114.0 (101.8)	93.7 (95.1)	89.5 (86.8)	99.4 (95.7)
Watching TV streaming platforms	110.3 (86.4)	111.0 (85.7)	122.4 (90.1)	109.4 (87.6)	97.7 (77.5)	110.5 (86.2)
Playing games	112.7 (98.9)	101.2 (89.8)	122.0 (101.0)	101.4 (94.4)	106.5 (94.0)	109.9 (96.9)
Browsing online, reading websites, Googling	44.5 (55.4)	68.2 (66.9)	53.8 (64.7)	45.6 (54.1)	52.3 (58.7)	50.3 (59.3)
Watching gaming or livestreaming on Twitch	25.7 (53.8)	45.9 (76.8)	36.1 (65.7)	26.7 (58.8)	29.2 (56.9)	30.6 (60.8)

^a^Parents were asked to select the racial categories that best described their child from the following options: Black; East Asian; South Asian; Southeast Asian; Indigenous; Latin American; Middle Eastern; White; other race category; and ‘prefer not to answer’. Participants indicating multiple races (Black, East Asian, South Asian, Southeast Asian, Indigenous, Latin American, Middle Eastern and/or White) were grouped into an ‘other or mixed’ race/ethnicity group category. Due to the limited number of observations in some racial/ethnic minority group categories, responses were combined into two broader categories: “White” and “racial/ethnic minority”, where the latter encompassed all responses except “White” and “prefer not to answer”

^b^After non-parametric Winsorization; values were weighted for weekdays (5/7) and weekend days (2/7)

### Self-reported exposure to digital food marketing

#### Frequency of exposure to digital food marketing

Fewer White youth were exposed to marketing of sugary drinks (62.7% vs. 76.5%), sugary cereals (48.1% vs. 66.7%), fruits or vegetables (27.8% vs. 39.5%), salty or savoury snacks (62.8% vs. 77.8%), fast foods (71.2% vs. 83.1%) and desserts or sweet treats (53.4% vs. 71.2%) via digital media than racial/ethnic minority participants in the past 7 days (Table 2; calculated as 100% minus the percentage reporting “never” having been exposed). Similarly, greater proportions of racial/ethnic minority participants reported exposure to each type of digital food marketing “1–3 times per week”, “4–6 times per week”, “every day” or “more than once a day” compared to participants identifying as White. Trends in the frequency of digital marketing exposure by income adequacy group were less consistent. A lower proportion of participants from households with medium income adequacy reported exposure to digital marketing of several types of foods, compared with participants of high or low income adequacy: sugary drinks (60.9% for medium income adequacy vs. 64.9% high vs. 72.8% low); sugary cereals (48.0% vs. 55.1% vs. 55.6%); fruits or vegetables (28.2% vs. 33.0% vs. 31.4%); fast foods (70.5% vs. 75.1% vs. 77.2%); and desserts or sweet treats (52.3% vs. 56.1% vs. 65.1%).

**Table 2:** The number and proportion of participants who reported being exposed to food marketing at various frequencies (*n* = 996)

Frequency of exposure to digital food marketing	Race/ethnicity		Income adequacy		
	White (*n* = 753)	Racial/ethnic minority (*n* = 243)	Low (*n* = 338)	Medium (*n* = 373)	High (*n* = 285)
	*n* (%)	*n* (%)	*n* (%)	*n* (%)	*n* (%)
Sugary drinks					
Never	281 (37.3)	57 (23.5)	92 (27.2)	146 (39.1)	100 (35.1)
1–3 times per week	323 (42.9)	116 (47.7)	159 (47.0)	164 (44.0)	116 (40.7)
4–6 times per week	63 (8.4)	23 (9.5)	32 (9.5)	23 (6.2)	31 (10.9)
Every day	61 (8.1)	38 (15.6)	39 (11.5)	31 (8.3)	29 (10.2)
More than once per day	25 (3.3)	9 (3.7)	16 (4.7)	9 (2.4)	9 (3.2)
Sugary cereals					
Never	391 (51.9)	81 (33.3)	150 (44.4)	194 (52.0)	128 (44.9)
1–3 times per week	247 (32.8)	98 (40.3)	118 (34.9)	127 (34.0)	100 (35.1)
4–6 times per week	58 (7.7)	31 (12.8)	35 (10.4)	24 (6.4)	30 (10.5)
Every day	41 (5.4)	27 (11.1)	25 (7.4)	22 (5.9)	21 (7.4)
More than once per day	16 (2.1)	6 (2.5)	10 (3.0)	6 (1.6)	6 (2.1)
Fruits or vegetables					
Never	544 (72.2)	147 (60.5)	232 (68.6)	268 (71.8)	191 (67.0)
1–3 times per week	135 (17.9)	58 (23.9)	64 (18.9)	77 (20.6)	52 (18.2)
4–6 times per week	40 (5.3)	16 (6.6)	20 (5.9)	13 (3.5)	23 (8.1)
Every day	26 (3.5)	18 (7.4)	16 (4.7)	12 (3.2)	16 (5.6)
More than once per day	8 (1.1)	4 (1.6)	6 (1.8)	3 (0.8)	3 (1.1)
Salty/savoury snacks					
Never	280 (37.2)	54 (22.2)	110 (32.5)	126 (33.8)	98 (34.4)
1–3 times per week	292 (38.8)	115 (47.3)	125 (37.0)	169 (45.3)	113 (39.6)
4–6 times per week	91 (12.1)	32 (13.2)	51 (15.1)	37 (9.9)	35 (12.3)
Every day	72 (9.6)	33 (13.6)	42 (12.4)	33 (8.8)	30 (10.5)
More than once per day	18 (2.4)	9 (3.7)	10 (3.0)	8 (2.1)	9 (3.2)
Fast foods					
Never	217 (28.8)	41 (16.9)	77 (22.8)	110 (29.5)	71 (24.9)
1–3 times per week	274 (36.4)	99 (40.7)	112 (33.1)	158 (42.4)	103 (36.1)
4–6 times per week	123 (16.3)	50 (20.6)	62 (18.3)	52 (13.9)	59 (20.7)
Every day	106 (14.1)	39 (16.0)	66 (19.5)	38 (10.2)	41 (14.4)
More than once per day	33 (4.4)	14 (5.8)	21 (6.2)	15 (4.0)	11 (3.9)
Desserts or sweet treats					
Never	351 (46.6)	70 (28.8)	118 (34.9)	178 (47.7)	125 (43.9)
1–3 times per week	252 (33.5)	111 (45.7)	129 (38.2)	139 (37.3)	95 (33.3)
4–6 times per week	68 (9.0)	26 (10.7)	39 (11.5)	24 (6.4)	31 (10.9)
Every day	57 (7.6)	31 (12.8)	39 (11.5)	22 (5.9)	27 (9.5)
More than once per day	25 (3.3)	5 (2.1)	13 (3.8)	10 (2.7)	7 (2.5)

The odds of more frequently being exposed to marketing of sugary drinks (OR: 0.70; 95% CI: 0.52, 0.94), sugary cereals (OR: 0.56; 95% CI: 0.42, 0.76), fruits and vegetables (OR: 0.63; 95% CI: 0.45, 0.87), salty or savoury snacks (OR: 0.63; 95% CI: 0.47, 0.85), fast food (OR: 0.74; 95% CI: 0.55, 0.99), and desserts or sweet treats (OR: 0.68; 95% CI: 0.50, 0.91) were lower for White participants than those of racial/ethnic minority groups (Table 3). Moreover, the odds of more frequently being exposed to marketing of sugary drinks (OR: 0.67; 95% CI: 0.51, 0.89), fast food (OR: 0.66; 95% CI: 0.50, 0.87), and desserts or sweet treats (OR: 0.65; 95% CI: 0.49, 0.87) were lower for participants from households of medium income adequacy than those of low income adequacy.

**Table 3:** Associations of race/ethnicity and income adequacy with self-reported frequency of digital food marketing exposure for different food categories (*n* = 996)^a, b^

	χ2, *p*-value	*p*-value	Adjusted OR (95% CI)
Sugary drinks			
Race/ethnicity	**5.61**	**0.02**	
White vs. racial/ethnic minority			**0.70 (0.52**,** 0.94)**
Income adequacy	**8.56**	**0.01**	
High vs. low			0.93 (0.69, 1.26)
Medium vs. low			**0.67 (0.51**,** 0.89)**
Sugary cereals			
Race/ethnicity	**14.06**	**< 0.001**	
White vs. racial/ethnic minority			**0.56 (0.42**,** 0.76)**
Income adequacy	5.79	0.06	
High vs. low			1.14 (0.84, 1.55)
Medium vs. low			0.80 (0.60, 1.06)
Fruits/vegetables			
Race/ethnicity	**7.64**	**0.006**	
White vs. racial/ethnic minority			**0.63 (0.45**,** 0.87)**
Income adequacy	4.80	0.09	
High vs. low			1.28 (0.91, 1.81)
Medium vs. low			0.89 (0.64, 1.23)
Salty or savoury snacks		**0.002**	
Race/ethnicity	**9.29**		
White vs. racial/ethnic minority			**0.63 (0.47**,** 0.85)**
Income adequacy	0.44	0.80	
High vs. low			1.05 (0.77, 1.42)
Medium vs. low			0.95 (0.72, 1.26)
Fast food			
Race/ethnicity	**4.17**	**0.04**	
White vs. racial/ethnic minority			**0.74 (0.55**,** 0.99)**
Income adequacy	**10.09**	**0.006**	
High vs. low			0.92 (0.69, 1.24)
Medium vs. low			**0.66 (0.50**,** 0.87)**
Dessert or sweet treats			
Race/ethnicity	**6.61**	**0.01**	
White vs. racial/ethnic minority			**0.68 (0.50**,** 0.91)**
Income adequacy	**8.81**	**0.01**	
High vs. low			0.85 (0.63, 1.15)
Medium vs. low			**0.65 (0.49**,** 0.87)**

^a^Proportional odds models were adjusted for age group, province, sex, BMI classification, device ownership, and total time spent on digital media per day (weighted), with race/ethnicity and income adequacy group as the independent variables of interest. Boldface indicates statistical significance at the alpha = 0.05 level. ^b^The reference category is listed second

#### Digital setting of exposure

The proportion of participants exposed to unhealthy food marketing on one or more social media platforms was greater for racial/ethnic minority youth (82.7%) than White participants (68.1%), and greater for participants from low income adequacy households (76.3%) than those of medium (70.5%) or high income adequacy (67.7%; Table 4). A similar trend was observed in the proportion of youth reporting exposure to unhealthy food marketing on at least one gaming, TV or music streaming platform/website where exposure was higher for racial/ethnic minority participants (49.8%) than those identifying as White (36.3%), and for participants of low income adequacy households (43.8%) compared to those of medium (37.8%) and high income adequacy (36.8%). White participants were less likely than racial/ethnic minority participants to report unhealthy food marketing exposure on one or more social media platform(s) (OR: 0.45; 95% CI: 0.30, 0.68) and at least one gaming, TV or music streaming platform(s)/website(s) (OR: 0.71; 95% CI: 0.51, 0.99; Table 5). There were no significant differences between income adequacy groups in terms of the odds of reporting exposure to unhealthy food marketing on one or more social media platform (χ2 = 2.15, *p* = 0.34) or gaming, TV or music streaming platform/website (χ2 = 0.79, *p* = 0.68).

**Table 4:** The number and proportion of participants who reported exposure to unhealthy food marketing on digital platforms and social media posts (*n* = 996)

Digital platform	Race/ethnicity		Income Adequacy		
	White (*n* = 753)	Racial/ethnic minority (*n* = 243)	Low (*n* = 338)	Medium (*n* = 373)	High (*n* = 285)
	*n* (%)	*n* (%)	*n* (%)	*n* (%)	*n* (%)
Social media^a^	513 (68.1)	201 (82.7)	258 (76.3)	263 (70.5)	193 (67.7)
Facebook	148 (19.7)	55 (22.6)	86 (25.4)	59 (15.8)	58 (20.4)
Instagram	152 (20.2)	78 (32.1)	93 (27.5)	59 (15.8)	78 (27.4)
X (formerly known as Twitter)	32 (4.2)	30 (12.3)	26 (7.7)	15 (4.0)	21 (7.4)
TikTok	251 (33.3)	85 (35.0)	134 (39.6)	104 (27.9)	98 (34.4)
Snapchat	89 (11.8)	50 (20.6)	49 (14.5)	43 (11.5)	47 (16.5)
YouTube	410 (54.4)	176 (72.4)	214 (63.3)	220 (59.0)	152 (53.3)
Pinterest	41 (5.4)	23 (9.5)	24 (7.1)	21 (5.6)	19 (6.7)
Blogs/websites	120 (15.9)	64 (26.3)	68 (20.1)	59 (15.8)	57 (20.0)
Posts and videos shared by influencers	181 (24.0)	68 (28.0)	109 (32.2)	83 (22.3)	57 (20.0)
Posts and video shared by friends on social media	145 (19.3)	64 (26.3)	85 (25.1)	64 (17.2)	60 (21.1)
Gaming, TV or music streaming platforms/websites^b^	273 (36.3)	121 (49.8)	148 (43.8)	141 (37.8)	105 (36.8)
Twitch	33 (4.4)	21 (8.6)	24 (7.1)	14 (3.8)	16 (5.6)
Gaming websites (e.g., Roblox)	122 (16.2)	51 (21.0)	74 (21.9)	49 (13.1)	50 (17.5)
Livestreamed games or eSports	48 (6.4)	35 (14.4)	34 (10.1)	18 (4.8)	31 (10.9)
TV streaming platforms (e.g., Netflix, Prime Video)	185 (24.6)	84 (34.6)	109 (32.2)	90 (24.1)	70 (24.6)
Spotify	62 (8.2)	44 (18.1)	39 (11.5)	39 (10.5)	28 (9.8)

^a^Includes participants reporting exposure to unhealthy food marketing on one or more social media platform(s) during the week before the survey. ^b^Includes participants reporting exposure to unhealthy food marketing on one or more gaming, TV or music streaming platform(s) or website(s) during the week before the survey

**Table 5:** Associations of race/ethnicity and income adequacy with self-reported unhealthy food marketing exposure via social media and gaming, TV or music streaming platforms/websites (*n* = 996).^a, b^

Type of digital platform	χ2	*p*-value	Adjusted OR (95% CI)
Social media			
Race/ethnicity	**14.65**	**< 0.001**	
White vs. racial/ethnic minority			**0.45 (0.30**,** 0.68)**
Income adequacy	2.15	0.34	
High vs. low			0.76 (0.53, 1.10)
Medium vs. low			0.84 (0.59, 1.19)
Gaming, TV or music streaming platforms/websites			
Race/ethnicity	**4.16**	**0.04**	
White vs. racial/ethnic minority			**0.71 (0.51**,** 0.99)**
Income adequacy	0.79	0.68	
High vs. low			0.88 (0.63, 1.24)
Medium vs. low			0.88 (0.64, 1.20)

^a^Logistic regression models were adjusted for age group, province, sex, BMI classification, device ownership, and total time spent on digital media per day (weighted), with race/ethnicity and income adequacy group as the independent variables of interest. Boldface indicates statistical significance at the alpha = 0.05 level. ^b^The reference category is listed second

#### Exposure to digital marketing techniques

The proportion of participants who were exposed to one or more marketing techniques in digital marketing of unhealthy food was greater for racial/ethnic minority participants (82.3%) than White participants (74.5%), and for participants of low income adequacy households (81.1%) than those of medium (75.3%) and high income adequacy (72.3%; Table 6). These trends were observed for several categories of marketing techniques: characters or child/teenage actors; child-appealing subjects, themes and language; celebrities, public figures and sports; visual design, audio and special effects; and incentives and premiums.

**Table 6:** The number and proportion of participants who reported being exposed to digital unhealthy food marketing that used each marketing technique (*n* = 996)

Marketing technique	Race/ethnicity		Income Adequacy		
	White (*n* = 753)	Racial/ethnic minority (*n* = 243)	Low (*n* = 338)	Medium (*n* = 373)	High (*n* = 285)
	*n* (%)	*n* (%)	*n* (%)	*n* (%)	*n* (%)
Exposed to one or more marketing techniques	561 (74.5)	200 (82.3)	274 (81.1)	281 (75.3)	206 (72.3)
Characters or child/teenage actors	346 (45.9)	138 (56.8)	180 (53.3)	177 (47.5)	127 (44.6)
Cartoon characters from movies or TV	158 (21.0)	70 (28.8)	87 (25.7)	84 (22.5)	57 (20.0)
Characters owned by food companies	204 (27.1)	93 (38.3)	117 (34.6)	108 (29.0)	72 (25.3)
Child or teenage actors	247 (32.8)	101 (41.6)	134 (39.6)	126 (33.8)	88 (30.9)
Subjects, themes and language	435 (57.8)	151 (62.1)	219 (64.8)	209 (56.0)	158 (55.4)
Themes like fun, popularity, being cool, being fashionable, being free and independent	356 (47.3)	127 (52.3)	179 (53.0)	174 (46.6)	130 (45.6)
Themes like magic, mystery, adventure or heroes	257 (34.1)	91 (37.4)	139 (41.1)	118 (31.6)	91 (31.9)
Slang used by children or teenagers	282 (37.5)	102 (42.0)	150 (44.4)	133 (35.7)	101 (35.4)
Celebrities, public figures and sports	416 (55.2)	142 (58.4)	215 (63.6)	195 (52.3)	148 (51.9)
Celebrities from movies, TV or bands	234 (31.1)	107 (44.0)	138 (40.8)	120 (32.2)	83 (29.1)
Social media influencers	206 (27.4)	87 (35.8)	117 (34.6)	102 (27.3)	74 (26.0)
Athletes, favourite sports teams	251 (33.3)	89 (36.6)	129 (38.2)	116 (31.1)	95 (33.3)
Extreme sports	236 (31.3)	79 (32.5)	117 (34.6)	111 (29.8)	87 (30.5)
Visual design, audio and special effects	501 (66.5)	169 (69.5)	246 (72.8)	248 (66.5)	176 (61.8)
Appealing bright colours	423 (56.2)	153 (63.0)	228 (67.5)	205 (55.0)	143 (50.2)
Catchy/popular music	373 (49.5)	113 (46.5)	177 (52.4)	180 (48.3)	129 (45.3)
Special effects/animation	272 (36.1)	104 (42.8)	147 (43.5)	137 (36.7)	92 (32.3)
References to product benefits, health or nutrition	424 (56.3)	154 (63.4)	214 (63.3)	201 (53.9)	163 (57.2)
New products	342 (45.4)	125 (51.4)	174 (51.5)	164 (44.0)	129 (45.3)
Saying the product is convenient	203 (27.0)	90 (37.0)	119 (35.2)	102 (27.3)	72 (25.3)
References to health or nutrition	226 (30.0)	98 (40.3)	130 (38.5)	106 (28.4)	88 (30.9)
Incentives and premiums	350 (46.5)	140 (57.6)	184 (54.4)	179 (48.0)	127 (44.6)
Contests	195 (25.9)	85 (35.0)	114 (33.7)	96 (25.7)	70 (24.6)
Free giveaways	160 (21.2)	76 (31.3)	96 (28.4)	78 (20.9)	62 (21.8)
Limited time offers	211 (28.0)	96 (39.5)	113 (33.4)	111 (29.8)	83 (29.1)
Price promotions	201 (26.7)	83 (34.2)	113 (33.4)	97 (26.0)	74 (26.0)
Reward/incentive programs	170 (22.6)	73 (30.0)	99 (29.3)	73 (19.6)	71 (24.9)
Cross-promotions	247 (32.8)	100 (41.2)	113 (33.4)	130 (34.9)	104 (36.5)
Link to a movie or TV show	188 (25.0)	85 (35.0)	88 (26.0)	106 (28.4)	79 (27.7)
Link to an event	149 (19.8)	59 (24.3)	69 (20.4)	73 (19.6)	66 (23.2)
Engagement techniques	261 (34.7)	99 (40.7)	137 (40.5)	126 (33.8)	97 (34.0)
Activities, polls, quizzes or games	141 (18.7)	63 (25.9)	75 (22.2)	67 (18.0)	62 (21.8)
Encouragement to like, comment or share with your friends on social media	218 (29.0)	85 (35.0)	125 (37.0)	105 (28.2)	73 (25.6)

^a^Includes participants reporting exposure to marketing of unhealthy foods using at least one of the marketing techniques from that category

White participants were less likely than racial/ethnic minority participants to report exposure to digital marketing of unhealthy food that featured techniques related to incentives and premiums (OR: 0.72; 95% CI: 0.518, 0.995) and cross-promotions (OR: 0.71; 95% CI: 0.51, 0.99; Table 7). Furthermore, compared with participants from households of low income adequacy, those of high (OR: 0.68; 95% CI: 0.49, 0.95) and medium (OR: 0.69; 95% CI: 0.50, 0.93) income adequacy households were less likely to report having been exposed to digital marketing featuring celebrities or public figures. No other significant differences in exposure to marketing techniques were observed between racial/ethnic and income adequacy groups. Similarly, the odds of being exposed to a greater number of marketing techniques for unhealthy foods did not differ between racial/ethnic (χ2 = 3.64, *p* = 0.06) or income adequacy groups (χ2 = 4.67, *p* = 0.10; results not shown).

**Table 7:** Associations of race/ethnicity and income adequacy with food marketing technique exposure in digital media (*n* = 996).^a, b^

Type of marketing technique	χ2	*p*-value	Adjusted OR (95% CI)
Characters or child/teenage actors			
Race/ethnicity	2.28	0.13	
White vs. racial/ethnic minority			0.77 (0.76, 1.29)
Income adequacy	0.81	0.67	
High vs. low			0.86 (0.62, 1.20)
Medium vs. low			0.92 (0.68, 1.26)
Subjects, themes and language			
Race/ethnicity	0.46	0.50	
White vs. racial/ethnic minority			0.89 (0.64, 1.25)
Income adequacy	3.39	0.18	
High vs. low			0.77 (0.55, 1.08)
Medium vs. low			0.76 (0.56, 1.05)
Celebrities and public figures			
Race/ethnicity	0.46	0.50	
White vs. racial/ethnic minority			0.89 (0.64, 1.24)
Income adequacy	**7.16**	**0.03**	
High vs. low			**0.68 (0.49**,** 0.95)**
Medium vs. low			**0.69 (0.50**,** 0.93)**
Visual design, audio and special effects			
Race/ethnicity	0.59	0.44	
White vs. racial/ethnic minority			0.87 (0.61, 1.24)
Income adequacy	4.80	0.09	
High vs. low			0.68 (0.48, 0.96)
Medium vs. low			0.80 (0.57, 1.11)
References to product benefits, health or nutrition			
Race/ethnicity	3.60	0.06	
White vs. racial/ethnic minority			0.72 (0.52, 1.01)
Income adequacy	4.48	0.11	
High vs. low			0.84 (0.60, 1.17)
Medium vs. low			0.72 (0.53, 0.98)
Incentives and premiums			
Race/ethnicity	**3.95**	**0.047**	
White vs. racial/ethnic minority			**0.718 (0.518**,** 0.995)**
Income adequacy	2.54	0.28	
High vs. low			0.77 (0.55, 1.06)
Medium vs. low			0.87 (0.64, 1.18)
Cross-promotions			
Race/ethnicity	**4.08**	**0.04**	
White vs. racial/ethnic minority			**0.71 (0.51**,** 0.99)**
Income adequacy	1.89	0.39	
High vs. low			1.27 (0.90, 1.79)
Medium vs. low			1.16 (0.84, 1.61)
Engagement techniques			
Race/ethnicity	0.58	0.45	
White vs. racial/ethnic minority			0.88 (0.63, 1.23)
Income adequacy	2.19	0.34	
High vs. low			0.84 (0.60, 1.18)
Medium vs. low			0.79 (0.58, 1.09)

^a^All models were adjusted for age group, province, sex, BMI classification, device ownership, and total time spent on digital media per day (weighted), with race/ethnicity and income adequacy group as the independent variables of interest. Boldface indicates statistical significance at the alpha = 0.05 level. ^b^The reference category is listed second

## Discussion

This study provides a novel examination of the associations of race/ethnicity and income adequacy with self-reported exposure to digital food marketing, particularly for unhealthy foods, in a sample of youth in Canada. Findings indicate that racial/ethnic minority youth were more frequently exposed to digital marketing of unhealthy foods and fruits and vegetables than those identifying as White. We also found some evidence that youth from lower income adequacy households were more frequently exposed to digital marketing of certain unhealthy foods relative to youth from medium income adequacy households; however, compared to race/ethnicity, this relationship was far less consistent across the measures examined. Moreover, no significant differences were observed relative to youth from high income adequacy households.

Results from this work align with the relatively small but consistent body of evidence which suggests that White children tend to be less exposed to unhealthy food marketing than those identifying as racial/ethnic minority groups [31– 36, 38]. While previous studies, mostly from the US, have shown that Black and Hispanic children are more exposed to unhealthy food marketing on television than their White counterparts [31–36], this is among the first studies to suggest that racial/ethnic minority youth may also be targeted in food marketing on digital media. In the present study, White youth were less likely than racial/ethnic minority youth to report more frequent exposure to marketing of all types of foods examined (sugary drinks and cereals, fruits/vegetables, salty/savoury snacks, fast food, and desserts/sweet treats), and to report exposure to unhealthy food marketing on one or more social media, gaming, TV or music streaming platforms/websites. These differences between racial/ethnic groups were observed independent of total time spent online per day and the number of digital devices owned. Such differences may therefore suggest that racialized youth are being targeted by unhealthy digital food marketing; however, this is unclear since racialized youth also saw more marketing for fruits and vegetables. It is therefore possible that they saw more marketing for all types of foods. Importantly, given the amalgamation of racial/ethnic minority participants into a single category, our study was not able to identify differences in marketing exposure between racial/ethnic minority groups. There is a need for future research to include larger samples of racial/ethnic minority youth, with analyses stratified by racial/ethnic minority group, to better explore differences in digital food marketing exposure.

It is well-established that digital marketing is tailored, personalized and targeted to individuals based on a large number and variety of data points, including sociodemographic information and browsing and clicking history, which are collected over time for all internet users, including youth [16, 73]. Recent research has estimated that more than 72 million data points are collected on a child by the time they turn 13 years old [73]. A Canadian study that examined the data collection practices of five fast food company applications reported that a variety of data were being collected on children aged 9–12 years, including sociodemographic data, food preferences and purchasing habits, frequency of app visits and in-app clicks, communications with other users, store ordering location and information about connection to in-store Wi-Fi, among others [74]. Additionally, a 2021 report by the US Federal Trade Commission found that many internet service providers collect copious amounts of personal data, including sensitive characteristics such as race/ethnicity [75]. Research has also suggested that food advertisements on US television target youth of racial/ethnic minority groups through the design and content of the ad (e.g., cultural themes, actors) [36]; such targeting likely occurs in digital marketing as well. Equally concerning is the lack of government regulations concerning information collected about youth online and the protection of their digital privacy [73, 74]. In Canada, the Personal Information Protection and Electronic Documents Act (PIPEDA) suggests that collection of data from children should be avoided or limited given that meaningful consent is challenging [76]; however, this is not strictly monitored or enforced and insufficiently protects children. In combination, this evidence suggests that the more frequent and powerful digital marketing of unhealthy foods reported by racial/ethnic minority youth in this study may reflect racially targeted marketing. However, additional research is needed, particularly in Canada, to better understand how internet users’ personal data are collected, used, and protected.

Consistent with previous research on this topic (mainly from the US), these findings provide some evidence of lower income adequacy youth being disproportionately exposed to food marketing, particularly for unhealthy foods, on digital media [31, 33, 35]. However, this study also suggests that income adequacy is a less important predictor of youth’s exposure to digital marketing of unhealthy foods than race/ethnicity. Youth from low income adequacy households were more likely than those of medium income adequacy to be more frequently exposed to digital marketing of sugary drinks, fast food, and desserts or sweet treats; there were, however, no differences between youth from households of low and high income adequacy in their frequency of exposure to digital marketing of all food categories examined. The lack of an observed negative association between income adequacy and marketing exposure as may have been expected was also observed in a similar Canadian study, where youth from households with ‘more than enough money’ reported more frequent food marketing exposure than those ‘with enough money’ [38]. The reasons for the differences in exposure between youth from low and middle income adequacy households – and the lack of differences between those from low and high income adequacy households – are unclear. They are unrelated to differences in screen time or digital device ownership, given the inclusion of these variables in the regression models, but may be linked to other digital device usage and lifestyle factors (e.g., use of ad blockers and/or paid apps or subscription streaming services without ads, parental supervision of digital media use). It is also possible that the total screen time variable included in the models did not fully capture differences in how participants used digital media, such as the digital platforms used and type of content consumed. It is also worth noting that condensing the income adequacy data into three categories may inadvertently mask subtle differences between youth of different household income adequacies. Further research to investigate why youth from middle-income homes tend to report lower levels of food marketing exposure is warranted.

Additionally, youth from low income adequacy households were more likely to report exposure to digital marketing of unhealthy foods that featured celebrities and public figures, compared with children and adolescents of medium or high income adequacy households. This is concerning, given that the use of celebrities in unhealthy food marketing has been shown to heighten product appeal and brand recognition, influence food preferences, and increase children’s consumption of unhealthy foods [77–81]. These findings reinforce a need for restrictions on the use of celebrities and public figures (e.g., actors, musicians, influencers, athletes) in unhealthy food marketing that appeal to youth. The Canadian government’s most recent policy proposal would restrict the use of celebrities and public figures that are popular with children. However, this restriction may have little impact given that most celebrities featured in food marketing appeal to both youth and adults (e.g., popular musicians, professional athletes) [44].

The differences in self-reported frequency and power of digital food marketing exposure between youth of different racial/ethnic groups and levels of household income adequacy may be more reflective of differences in marketing recall than their actual exposure. For instance, racial/ethnic minority youth may have been more likely to recall marketing instances featuring actors of similar races/ethnicities [82, 83]. Moreover, television advertising has been shown to have more influence on food choice among adults with greater cognitive loads (e.g., due to stress, discrimination, negative emotions and/or daily pressures), with the authors suggesting that people of racial/ethnic minority groups and lower socioeconomic status have higher cognitive loads and may therefore be more susceptible to food advertising [84]. Racial/ethnic minority youth and those from low income adequacy households in this study may therefore have recalled more frequent and/or powerful exposure to digital food marketing in part due to higher cognitive loads associated with their racial/ethnic identity and/or household income status. Our results may also reflect differences between racial/ethnic and income groups in the foods they consider to be healthy and the digital content they consider to be marketing [85].

Findings from this study suggest that self-regulatory initiatives (i.e., the CCFBA, CAI) are failing to sufficiently protect youth in Canada from vulnerable population subgroups (racial/ethnic minorities and low income adequacy households) from unhealthy food marketing on digital media, and support the need for federal regulations to reduce their exposure. The federal regulations being considered in Canada only propose to restrict unhealthy food marketing on television and digital media (including websites, social media, mobile apps, broadcast television, email and messaging services, video and audio streaming services, and online games and virtual reality programs) targeted at children under 13 years of age [44]. Such regulations may have limited impact given that much of the marketing on digital media would not be considered “child-directed” due to its broad appeal to individuals of all ages and that adolescents will not be protected. If the federal government does restrict food marketing to children, it will be important to monitor its impact on racialized and socioeconomically disadvantaged children (and adolescents) and ensure that all children (including adolescents aged 13–17 years) are equally protected. Moreover, these findings suggest that Quebec’s Consumer Protection Act [43] may be failing to protect children from digital marketing of unhealthy foods. This may, at least in part, be related to the emergence of new digital media platforms, increases in children’s digital media use, the evolution of novel digital marketing techniques since the guide for the application of the Consumer Protection Act was last published in 2012 and/or a lack of monitoring [43]. However, children who were from Quebec and under 13 years of age (and therefore protected under the Consumer Protection Act) only constituted about one-quarter of the sample and subgroup analyses by age and province were beyond the scope of this study. Previously published work by our group based on these same survey data examined differences in self-reported exposure to digital marketing of unhealthy foods between children and adolescents in Ontario and Quebec [53].

### Strengths and limitations

This study provides a novel comparison of youth’s exposure to digital marketing of unhealthy foods across racial/ethnic and income groups. It is strengthened by its examination of marketing on several digital platforms popular among youth, and by adjusting for participants’ sociodemographic characteristics and habitual digital media usage in the analyses. Nonetheless, this study did not measure differences between racial/ethnic and income adequacy groups in terms of absolute volume of exposures to unhealthy food marketing via each digital platform and marketing technique. Examining absolute exposure may have altered some of our results, particularly for income adequacy, where few differences in the proportion of participants exposed to unhealthy food marketing or marketing techniques were observed between groups (after adjusting for covariates). For example, a UK study found that despite no difference in proportional exposure (i.e., the proportion of all marketing exposures that were for unhealthy food) to unhealthy food marketing on television by children of households from different income brackets, children from less affluent families had a higher absolute exposure to unhealthy food marketing than their more affluent counterparts. Future studies should consider both proportional and absolute exposure (e.g., by examining the number of times youth were exposed to marketing via each digital platform or marketing technique) [86].

In addition, the data presented in this study was self-reported by youth, which may be subject to misreporting (e.g., recall error, misinterpretation) and to systematic between-group differences in perceptions of what constituted unhealthy foods and marketing. Specifically, children aged 10–12 years may have struggled to identify or recall instances of advertisements on digital media, or to differentiate ads from other content. There may also be systematic bias in recall between different racial/ethnic and/or income adequacy subgroups. For example, youth of racial/ethnic minority groups may be more likely to identify and recall advertisements that were culturally relevant to them [87]. Recall errors, misinterpretations or biases may have made differences in marketing exposure between racial/ethnic and/or income adequacy groups appear greater than they are. Furthermore, this study only asked about exposure over the previous 7 days at a single point in time, which may not be representative of the sampled youth’s typical digital marketing exposure. Nonetheless, food and tobacco marketing studies have indicated that self-reported data is a valid measure of digital marketing exposure (and exposure on television, in stores and in outdoor settings) [88, 89]. Self-reported food marketing exposure measures have also been used in numerous previous studies involving large population samples of youth aged 10–17 years [38, 53, 54, 59]^90^– [93]. Additionally, compared with objective measures of marketing exposure (e.g., studies using screen capture methodology), self-reported data are much less expensive and labour-intensive to collect, thereby enabling more frequent monitoring of youth’s exposure to unhealthy food marketing, which is critical for informing proposed and existing policies concerning food marketing to youth [38].

Importantly, the nonprobability-based sampling strategy for this study was not conducive to generating a representative sample, which may limit the generalizability of our findings. Specifically, participants were only recruited from two Canadian provinces (Ontario and Quebec); youth from other provinces or territories may have reported different levels of exposure. It is also worth noting that our sample excludes youth without a digital device to complete the survey. These youth are presumably less exposed to digital food marketing than those with digital devices, thereby potentially overestimating digital marketing exposure among Canadian youth. Generalizability may also be hindered by the fact that our sample was largely comprised of White participants (76%), as is common in survey panels such as Leger [94]. This does, however, align with the national population estimate, with approximately 74% of Canadians identifying as White according to Canada’s 2021 census [95]. Nonetheless, some racial/ethnic minority groups may have been underrepresented in our sample. Similarly, although about 29% of our sample identified reported have high income adequacy, this aligns with national estimates, with 23% of Canadians reporting annual incomes of at least $100,000 as of 2022 [96]. Furthermore, although participants were asked to specify their races/ethnicities, those identifying as racial/ethnic minority groups were collapsed into a single category due to relatively small numbers of observations in each individual category, resulting in limited information about differences in marketing exposure between racial/ethnic groups. In addition, the exclusion of 215 participants because of missing data, which may have biased our findings if these individuals were systematically different or represented specific racial/ethnic or income groups. Other limitations include the potential for residual confounding, the lack of adjustment for multiple comparisons, and the fact that this was a cross-sectional study conducted at one point in time and thus, causality cannot be established. Lastly, some participants may have had ad blockers on their devices, thereby potentially limiting their exposure to food marketing on digital media.

## Conclusions

In conclusion, this research found that digital food marketing exposure, particularly for unhealthy foods, is generally higher for Canadian youth of racial/ethnic minority groups than those identifying as White. While we found less evidence of differences between income adequacy groups, some differences in marketing exposure were observed for certain food categories and marketing techniques. These findings suggest a need for regulations to limit all youths’ exposure to unhealthy food marketing across various digital platforms. Future research is needed to build a stronger evidence base concerning differences in youths’ unhealthy food marketing exposure by race/ethnicity and income groups on digital media and in other settings.

## Supplementary Information

Below is the link to the electronic supplementary material.

Supplementary Material 1

## Data Availability

Data generated or analyzed during this study are available from the corresponding author upon reasonable request.

## Abbreviations

BMI: Body Mass Index
CAI: Canadian Children’s Food and Beverage Advertising Initiative
CCFBA: Code for the Responsible Advertising of Food and Beverage Products to Children
PIPEDA: The Personal Information Protection and Electronic Documents Act
WHO: World Health Organization

## References

[1] Globan Burden of Disease Collaborators. Health effects of dietary risks in 195 countries, 1990–2017: a systematic analysis for the global burden of disease study 2017. Lancet. 2019;393(10184):1958–72. 30954305 10.1016/S0140-6736(19)30041-8 PMC6899507

[2] Garriguet D. Diet quality in Canada. Health Rep. 2009;20(3):41–52. 19813438

[3] Jessri M, Nishi SK, L’Abbé MR. Assessing the nutritional quality of diets of Canadian adults using the 2014 health Canada surveillance tool tier system. Nutrients. 2015;7(12):10447–68. 26703721 10.3390/nu7125543 PMC4690095

[4] Hack S, Jessri M, L’Abbé MR. Nutritional quality of the food choices of Canadian children. BMC Nutr. 2021;7(1):16. 34049592 10.1186/s40795-021-00422-6 PMC8164219

[5] Tugault-Lafleur CN, Black JL. Differences in the quantity and types of foods and beverages consumed by Canadians between 2004 and 2015. Nutrients. 2019;11(3). 10.3390/nu11030526 PMC6471131 30823448

[6] Thomson JL, Tussing-Humphreys LM, Goodman MH, Landry AS. Diet quality in a nationally representative sample of American children by sociodemographic characteristics. Am J Clin Nutr. 2019;109(1):127–38. 30596813 10.1093/ajcn/nqy284

[7] Kirkpatrick SI, Dodd KW, Reedy J, Krebs-Smith SM. Income and race/ethnicity are associated with adherence to food-based dietary guidance among US adults and children. J Acad Nutr Diet. 2012;112(5):624–e356. 22709767 10.1016/j.jand.2011.11.012 PMC3775640

[8] Ng SW, Poti JM, Popkin BM. Trends in racial/ethnic and income disparities in foods and beverages consumed and purchased from stores among US households with children, 2000–2013. Am J Clin Nutr. 2016;104(3):750–9. 27488233 10.3945/ajcn.115.127944 PMC4997294

[9] Hutchinson J, Tarasuk V. The relationship between diet quality and the severity of household food insecurity in Canada. Public Health Nutr. 2022;25(4):1013–26. 34551845 10.1017/S1368980021004031 PMC9991759

[10] Olstad DL, Nejatinamini S, Victorino C, Kirkpatrick SI, Minaker LM, McLaren L. Trends in socioeconomic inequities in diet quality between 2004 and 2015 among a nationally representative sample of children in Canada. J Nutr. 2021;151(12):3781–94. 34515311 10.1093/jn/nxab297 PMC8643615

[11] Riediger ND, LaPlante J, Mudryj A, Clair L. Diet quality among Indigenous and non-Indigenous children and youth in Canada in 2004 and 2015: a repeated cross-sectional design. Public Health Nutr. 2022;25(1):123–32. 34108071 10.1017/S1368980021002561 PMC8825962

[12] Hiza HA, Casavale KO, Guenther PM, Davis CA. Diet quality of Americans differs by age, sex, race/ethnicity, income, and education level. J Acad Nutr Diet. 2013;113(2):297–306. 23168270 10.1016/j.jand.2012.08.011

[13] Calvert SL. Children as consumers: advertising and marketing. Future Child. 2008;18(1):205–34. 21338011 10.1353/foc.0.0001

[14] Smith R, Kelly B, Yeatman H, Boyland E. Food Marketing Influences Children’s Attitudes, Preferences and Consumption: A Systematic Critical Review. Nutrients. 2019;11(4). 10.3390/nu11040875 PMC6520952 31003489

[15] World Health Organization. Policies to protect children from the harmful impact of food marketing: WHO guideline 2023. https://www.who.int/publications/i/item/9789240075412. Accessed 27 May 2025. 37440677

[16] Boyland E, Thivel D, Mazur A, Ring-Dimitriou S, Frelut ML, Weghuber D, et al. Digital food marketing to young people: A substantial public health challenge. Ann Nutr Metab. 2020;76(1):6–9. 32101856 10.1159/000506413

[17] Brisson-Boivin K. The Digital Well-Being of Canadian Families: MediaSmarts; 2018. https://mediasmarts.ca/sites/mediasmarts/files/publication-report/full/digital-canadian-families.pdf. Accessed 27 May 2025.

[18] Statista. April. Mobile phone usage among children and teens in Canada as of 2022, by age group 2022. https://www.statista.com/statistics/1319950/canada-mobile-usage-kids-and-teens-by-age/#:~:text=As%20of%20April%202022%2C%2039,between%2012%20and%2017%20years. Accessed 27 May 2025.

[19] Potvin Kent M, Pauzé E, Bagnato M, Guimarães JS, Pinto A, Remedios L, et al. Food and beverage advertising expenditures in Canada in 2016 and 2019 across media. BMC Public Health. 2022;22(1):1458. 35915428 10.1186/s12889-022-13823-4 PMC9340686

[20] Pew Research Center, Teens, Social Media and Technology. 2023. 2023. https://www.pewresearch.org/internet/2023/12/11/teens-social-media-and-technology-2023/. Accessed 27 May 2025.

[21] Common Sense Media. The Common Sense Census: Media Use by Tweens and Teens. 2021, Media Use by Children in Lower-Income Households. 2022. https://www.commonsensemedia.org/sites/default/files/research/report/2021-8-18-census-fact-sheet-media-use-by-children-in-lower-income-households_0.pdf. Accessed 27 May 2025.

[22] Matz SC, Kosinski M, Nave G, Stillwell DJ. Psychological targeting as an effective approach to digital mass persuasion. Proc Natl Acad Sci U S A. 2017;114(48):12714–9. 29133409 10.1073/pnas.1710966114 PMC5715760

[23] World Health Organization. Food marketing exposure and power and their associations with food-related attitudes, beliefs and behaviours: a narrative review. 2022. https://www.who.int/publications/i/item/9789240041783. Accessed 27 May 2025.

[24] Potvin Kent M, Hatoum F, Wu D, Remedios L, Bagnato M. Benchmarking unhealthy food marketing to children and adolescents in canada: a scoping review. Health Promot Chronic Dis Prev Can. 2022;42(8):307–18. 35993602 10.24095/hpcdp.42.8.01 PMC9514213

[25] Amson A, Pauzé E, Remedios L, Pritchard M, Potvin Kent M. Adolescent exposure to food and beverage marketing on social media by gender: a pilot study. Public Health Nutr. 2023;26(1):33–45. 36321517 10.1017/S1368980022002312 PMC11077454

[26] Potvin Kent M, Pauzé E. The frequency and healthfulness of food and beverages advertised on adolescents’ preferred web sites in Canada. J Adolesc Health. 2018;63(1):102–7. 30060846 10.1016/j.jadohealth.2018.01.007

[27] Potvin Kent M, Pauzé E, Roy EA, de Billy N, Czoli C. Children and adolescents’ exposure to food and beverage marketing in social media apps. Pediatr Obes. 2019;14(6):e12508. 30690924 10.1111/ijpo.12508 PMC6590224

[28] Potvin Kent M, Pauzé E. The effectiveness of self-regulation in limiting the advertising of unhealthy foods and beverages on children’s preferred websites in Canada. Public Health Nutr. 2018;21(9):1608–17. 29433594 10.1017/S1368980017004177 PMC10261474

[29] Kent MP, Dubois L, Kent EA, Wanless AJ. Internet marketing directed at children on food and restaurant websites in two policy environments. Obes (Silver Spring). 2013;21(4):800–7. 10.1002/oby.20124 23712983

[30] Vergeer L, Vanderlee L, Potvin Kent M, Mulligan C, L’Abbé MR. The effectiveness of voluntary policies and commitments in restricting unhealthy food marketing to Canadian children on food company websites. Appl Physiol Nutr Metab. 2019;44(1):74–82. 30273499 10.1139/apnm-2018-0528

[31] Backholer K, Gupta A, Zorbas C, Bennett R, Huse O, Chung A, et al. Differential exposure to, and potential impact of, unhealthy advertising to children by socio-economic and ethnic groups: A systematic review of the evidence. Obes Rev. 2021;22(3):e13144. 33073488 10.1111/obr.13144

[32] Fleming-Milici F, Harris JL. Television food advertising viewed by preschoolers, children and adolescents: contributors to differences in exposure for black and white youth in the united States. Pediatr Obes. 2018;13(2):103–10. 27977909 10.1111/ijpo.12203

[33] Coleman PC, Hanson P, van Rens T, Oyebode O. A rapid review of the evidence for children’s TV and online advertisement restrictions to fight obesity. Prev Med Rep. 2022;26:101717. 35141122 10.1016/j.pmedr.2022.101717 PMC8814640

[34] Powell LM, Szczypka G, Chaloupka FJ. Adolescent exposure to food advertising on television. Am J Prev Med. 2007;33(4 Suppl):S251–6. 17884573 10.1016/j.amepre.2007.07.009

[35] Powell LM, Wada R, Kumanyika SK. Racial/ethnic and income disparities in child and adolescent exposure to food and beverage television Ads across the U.S. Media markets. Health Place. 2014;29:124–31. 25086271 10.1016/j.healthplace.2014.06.006 PMC4501500

[36] Harris JL, Fleming-Milici F, Mancini S, Kumanyika S, Ramirez AG. Rudd Report: Targeted food and beverage advertising to Black and Hispanic consumers: 2022 update. 2022. https://uconnruddcenter.org/wp-content/uploads/sites/2909/2022/11/Rudd-Targeted-Marketing-Report-2022.pdf. Accessed 27 May 2025.

[37] Velazquez CE, Black JL, Potvin Kent M. Food and beverage marketing in schools: A review of the evidence. Int J Environ Res Public Health. 2017;14(9). 10.3390/ijerph14091054 PMC5615591 28895921

[38] Acton RB, Bagnato M, Remedios L, Potvin Kent M, Vanderlee L, White CM, et al. Examining differences in children and adolescents’ exposure to food and beverage marketing in Canada by sociodemographic characteristics: findings from the international food policy study youth survey, 2020. Pediatr Obes. 2023;18(6):e13028. 36958860 10.1111/ijpo.13028

[39] Statista. Share of children with an electronic device in their home in the United States in 2019 and 2021, by type. 2023. https://www.statista.com/statistics/1324245/children-owning-an-electronic-device-at-home-by-type-us/#:~:text=During%20a%202021%20survey%20conducted,percent%20recorded%20two%20years%20before. Accessed 27 May 2025.

[40] Madigan S, Eirich R, Pador P, McArthur BA, Neville RD. Assessment of changes in child and adolescent screen time during the COVID-19 pandemic: A systematic review and Meta-analysis. JAMA Pediatr. 2022;176(12):1188–98. 36342702 10.1001/jamapediatrics.2022.4116 PMC9641597

[41] Ad Standards. Code for the Responsible Advertising of Food and Beverage Products to Children. 2023. https://adstandards.ca/resources/library/code-responsible-advertising-food-beverage-products-to-children/. Accessed 27 May 2025.

[42] Boyland E, McGale L, Maden M, Hounsome J, Boland A, Jones A. Systematic review of the effect of policies to restrict the marketing of foods and non-alcoholic beverages to which children are exposed. Obes Rev. 2022;23(8):e13447. 35384238 10.1111/obr.13447 PMC9541016

[43] Office de la protection du consommateur. Advertising Directed at Children under 13 Years of Age: Guide to the Application of Sects. 248 and 249 Consumer Protection Act. 2012.

[44] Government of Canada. Policy update on restricting food advertising primarily directed at children: Overview 2023 [updated June 20, 2023]. https://www.canada.ca/en/health-canada/services/food-nutrition/healthy-eating-strategy/policy-update-restricting-food-advertising-primarily-directed-children.html. Accessed 27 May 2025.

[45] Statistics Canada. The Canadian census: A rich portrait of the country’s religious and ethnocultural diversity. 2022. https://www150.statcan.gc.ca/n1/daily-quotidien/221026/dq221026b-eng.htm. Accessed 27 May 2025.

[46] Statistics Canada, Canadian Income Survey. 2021. 2023. https://www150.statcan.gc.ca/n1/en/daily-quotidien/230502/dq230502a-eng.pdf?st=7VVK4Nbs. Accessed 27 May 2025.

[47] Statistics Canada, Canadian Income Survey. 2022. 2024. https://www150.statcan.gc.ca/n1/daily-quotidien/240426/dq240426a-eng.htm. Accessed 27 May 2025.

[48] Hammond D. International Food Policy Study: Methods. https://foodpolicystudy.com/methods/. Accessed 27 May 2025.

[49] Fleming-Milici F, Harris JL. Adolescents’ engagement with unhealthy food and beverage brands on social media. Appetite. 2020;146:104501. 31669579 10.1016/j.appet.2019.104501

[50] Baldwin HJ, Freeman B, Kelly B. Like and share: associations between social media engagement and dietary choices in children. Public Health Nutr. 2018;21(17):3210–5. 30086811 10.1017/S1368980018001866 PMC10260990

[51] Leger Marketing Inc. Leger 2023. https://leger360.com/. Accessed 27 May 2025.

[52] Quest Mindshare. Services. 2021. https://questmindshare.com/services/#Panel. Accessed 27 May 2025.

[53] Vergeer L, Soto C, Bagnato M, Pauzé E, Amson A, Ramsay T, et al. Examining differences in exposure to digital marketing of unhealthy foods reported by Canadian children and adolescents in two policy environments. BMC Nutr. 2025;11(1):32. 39920856 10.1186/s40795-025-01019-z PMC11806838

[54] Vergeer L, Soto C, Bagnato M, Pauzé E, Amson A, Ramsay T, et al. The relationship between youth’s exposure to unhealthy digital food marketing and their dietary intake in Canada. Appl Physiol Nutr Metab. 2024;49(12):1678–91. 39190934 10.1139/apnm-2024-0224

[55] Ad Standards. Children’s Food and Beverage Advertising Initiative. http://www.adstandards.com/en/childrensinitiative/default.htm. Accessed 27 May 2025.

[56] Senate of Canada. C-252: An Act to amend the Food and Drugs Act (prohibition of food and beverage marketing directed at children). 2023. https://www.parl.ca/legisinfo/en/bill/44-1/c-252. Accessed 27 May 2025.

[57] World Health Organization. BMI-for-age (5–19 years). https://www.who.int/tools/growth-reference-data-for-5to19-years/indicators/bmi-for-age. Accessed 27 May 2025.

[58] Acton R, White C, Rynard V, Hammond D. Perceived income adequacy versus household income as a measure of socioeconomic status in cross-sectional population-level surveys conducted in six countries: an analysis of the 2022–2023 international food policy study. Public Health Reports: In; 2025. 10.1177/00333549251358655 PMC12367702 40832816

[59] Demers-Potvin É, White M, Potvin Kent M, Nieto C, White CM, Zheng X, et al. Adolescents’ media usage and self-reported exposure to advertising across six countries: implications for less healthy food and beverage marketing. BMJ Open. 2022;12(5):e058913. 35589343 10.1136/bmjopen-2021-058913 PMC9121490

[60] Acton RB, Vanderlee L, Cameron AJ, Goodman S, Jáuregui A, Sacks G, et al. Self-Reported impacts of the COVID-19 pandemic on Diet-Related behaviors and food security in 5 countries: results from the international food policy study 2020. J Nutr. 2022;152(Suppl 1):S35–46. 10.1093/jn/nxac025 PMC8992246 35274699

[61] Kwon J, Cameron AJ, Hammond D, White CM, Vanderlee L, Bhawra J, et al. A multi-country survey of public support for food policies to promote healthy diets: findings from the international food policy study. BMC Public Health. 2019;19(1):1205. 31477071 10.1186/s12889-019-7483-9 PMC6721115

[62] Gómez-Donoso C, Sacks G, Vanderlee L, Hammond D, White CM, Nieto C, et al. Public support for healthy supermarket initiatives focused on product placement: a multi-country cross-sectional analysis of the 2018 international food policy study. Int J Behav Nutr Phys Act. 2021;18(1):78. 34127002 10.1186/s12966-021-01149-0 PMC8201822

[63] Bhawra J, Kirkpatrick SI, Hall MG, Vanderlee L, White CM, Hammond D. Patterns and correlates of nutrition knowledge across five countries in the 2018 international food policy study. Nutr J. 2023;22(1):19. 36922823 10.1186/s12937-023-00844-x PMC10018957

[64] Yau A, Adams J, Boyland EJ, Burgoine T, Cornelsen L, de Vocht F, et al. Sociodemographic differences in self-reported exposure to high fat, salt and sugar food and drink advertising: a cross-sectional analysis of 2019 UK panel data. BMJ Open. 2021;11(4):e048139. 33827849 10.1136/bmjopen-2020-048139 PMC8031692

[65] Bhawra J, Kirkpatrick SI, Hall MG, Vanderlee L, Thrasher JF, Hammond D. Correlates of Self-Reported and functional Understanding of nutrition labels across 5 countries in the 2018 international food policy study. J Nutr. 2022;152(Suppl 1):S13–24. 10.1093/jn/nxac018 PMC9188861 35274701

[66] Pew Research Center. The Demographics of Device Ownership. 2015. https://www.pewresearch.org/internet/2015/10/29/the-demographics-of-device-ownership/. Accessed 27 May 2025.

[67] Nieto C, Espinosa F, Valero-Morales I, Boyland E, Potvin Kent M, Tatlow-Golden M, et al. Digital food and beverage marketing appealing to children and adolescents: an emerging challenge in Mexico. Pediatr Obes. 2023;18(7):e13036. 37078451 10.1111/ijpo.13036

[68] Boyland E, Muc M, Coates A, Ells L, Halford JCG, Hill Z, et al. Food marketing, eating and health outcomes in children and adults: a systematic review and meta-analysis. Br J Nutr. 2025;133(6):781–805. 40518855 10.1017/S0007114524000102 PMC12169957

[69] Nagata JM, Ganson KT, Iyer P, Chu J, Baker FC, Pettee Gabriel K et al. Sociodemographic correlates of contemporary screen time use among 9- and 10-Year-Old children. J Pediatr. 2022;240:213 – 20.e2. 10.1016/j.jpeds.2021.08.077 PMC9107378 34481807

[70] Liang R, Goto R, Okubo Y, Rehkopf DH, Inoue K. Poverty and childhood obesity: current evidence and methodologies for future research. Curr Obes Rep. 2025;14(1):33. 40210845 10.1007/s13679-025-00627-x PMC11985678

[71] Kim H, Rajbhandari A, Krile R, Lang IM, Antonakos CL, Colabianchi N. Body mass index trajectories among the healthy communities study children: racial/ethnic and socioeconomic disparities in childhood obesity. J Racial Ethn Health Disparities. 2024;11(1):203–15. 36656440 10.1007/s40615-023-01511-x PMC9851105

[72] Harrell F. Violation of Proportional Odds is Not Fatal: Statistical Thinking; 2020. https://www.fharrell.com/post/po/. Accessed 17 Jul 2025.

[73] World Health Organization Regional Office for Europe. Tackling food marketing to children in a digital world: trans-disciplinary perspectives. 2016. http://www.euro.who.int/__data/assets/pdf_file/0017/322226/Tackling-food-marketing-children-digital-world-trans-disciplinary-perspectives-en.pdf. Accessed 27 May 2025.

[74] Potvin Kent M, Mulligan C, Gillis G, Remedios L, Parsons C. Restaurant Apps and Children’s Privacy: Exploring how children’s data and privacy are being protected on the mobile applications of top Canadian fast food and dine-in restaurants.: Heart & Stroke Foundation of Canada; 2023. https://www.heartandstroke.ca/-/media/pdf-files/advocacy/monique-potvin-kent-privacy-report-en.pdf?rev=28a626b372bf45bcac5bee0ddfcf5ae8. Accessed 27 May 2025.

[75] Federal Trade Commission. A Look at What ISPs Know About You: Examining the Privacy Practices of Six Major Internet Service Providers. 2021. https://www.ftc.gov/reports/look-what-isps-know-about-you-examining-privacy-practices-six-major-internet-service-providers. Accessed 27 May 2025.

[76] Office of the Privacy Commissioner of Canada. Guidelines for obtaining meaningful consent. 2021. https://www.priv.gc.ca/en/privacy-topics/collecting-personal-information/consent/gl_omc_201805/#_consent. Accessed 27 May 2025.

[77] Packer J, Russell SJ, Siovolgyi G, McLaren K, Stansfield C, Viner RM et al. The impact on dietary outcomes of celebrities and influencers in marketing unhealthy foods to children: A systematic review and Meta-Analysis. Nutrients. 2022;14(3). 10.3390/nu14030434 PMC8837952 35276800

[78] Calvo-Porral C, Rivaroli S, Orosa-González J. The influence of celebrity endorsement on food consumption behavior. Foods. 2021;10(9). 10.3390/foods10092224 PMC8471056 34574334

[79] Bergkvist L, Zhou KQ. Celebrity endorsements: a literature review and research agenda. Int J Advertising. 2016;35(4):642–63.

[80] Russell CA. Uncovering the power of natural endorsements: a comparison with celebrity-endorsed advertising and product placements. Int J Advertising. 2017;36(5):761–78.

[81] Boyland EJ, Harrold JA, Dovey TM, Allison M, Dobson S, Jacobs MC, et al. Food choice and overconsumption: effect of a premium sports celebrity endorser. J Pediatr. 2013;163(2):339–43. 23490037 10.1016/j.jpeds.2013.01.059

[82] Harris JL, Brownell KD, Bargh JA. The food marketing defense model: integrating psychological research to protect youth and inform public policy. Soc Issues Policy Rev. 2009;3(1):211–71. 20182647 10.1111/j.1751-2409.2009.01015.x PMC2826802

[83] Bragg MA, Miller AN, Kalkstein DA, Elbel B, Roberto CA. Evaluating the influence of Racially targeted food and beverage advertisements on black and white adolescents’ perceptions and preferences. Appetite. 2019;140:41–9. 31055011 10.1016/j.appet.2019.05.001 PMC9743992

[84] Zimmerman FJ, Shimoga SV. The effects of food advertising and cognitive load on food choices. BMC Public Health. 2014;14:342. 24721289 10.1186/1471-2458-14-342 PMC4021209

[85] Ojo AS, Nnyanzi LA, Giles EL, Ells LJ, Awolaran O, Okeke SR, et al. Perceptions of dietary intake amongst black, Asian and other minority ethnic groups in high-income countries: a systematic review of qualitative literature. BMC Nutr. 2023;9(1):85. 37443077 10.1186/s40795-023-00743-8 PMC10339484

[86] Adams J, Tyrrell R, Adamson AJ, White M. Socio-economic differences in exposure to television food advertisements in the UK: a cross-sectional study of advertisements broadcast in one television region. Public Health Nutr. 2012;15(3):487–94. 21806864 10.1017/S1368980011001686

[87] Miller A, Cassidy O, Greene T, Arshonsky J, Albert SL, Bragg MA. A Qualitative Analysis of Black and White Adolescents’ Perceptions of and Responses to Racially Targeted Food and Drink Commercials on Television. Int J Environ Res Public Health. 2021;18(21). 10.3390/ijerph182111563 PMC8583111 34770078

[88] Southwell BG, Barmada CH, Hornik RC, Maklan DM. Can we measure encoded exposure? Validation evidence from a National campaign. J Health Commun. 2002;7(5):445–53. 12455763 10.1080/10810730290001800 PMC4133105

[89] Scott LJ, Toumpakari Z, Nobles J, Sillero-Rejon C, Jago R, Cummins S, et al. Assessing exposure to outdoor advertisement for products high in fat, salt and sugar (HFSS); is self-reported exposure a useful exposure metric? BMC Public Health. 2023;23(1):668. 37041569 10.1186/s12889-023-15567-1 PMC10088263

[90] Bagnato M, Roy-Gagnon MH, Vanderlee L, White C, Hammond D, Potvin Kent M. The impact of fast food marketing on brand preferences and fast food intake of youth aged 10–17 across six countries. BMC Public Health. 2023;23(1):1436. 37501119 10.1186/s12889-023-16158-w PMC10373354

[91] Remedios L, Roy-Gagnon MH, Vanderlee L, Hammond D, Kent MP. The impact of exposure to sugary drink marketing on youth brand preference and recall: a cross-sectional and multi-country analysis. BMC Public Health. 2024;24(1):3275. 39592972 10.1186/s12889-024-20770-9 PMC11590244

[92] Scully M, Wakefield M, Niven P, Chapman K, Crawford D, Pratt IS, et al. Association between food marketing exposure and adolescents’ food choices and eating behaviors. Appetite. 2012;58(1):1–5. 22001023 10.1016/j.appet.2011.09.020

[93] Kumar G, Onufrak S, Zytnick D, Kingsley B, Park S. Self-reported advertising exposure to sugar-sweetened beverages among US youth. Public Health Nutr. 2015;18(7):1173–9. 25166512 10.1017/S1368980014001785 PMC10271747

[94] Wang-Schweig M, Miller BA, Buller DB, Byrnes HF, Bourdeau B, Rogers V. Using panel vendors for recruitment into a Web-Based family prevention program: methodological considerations. Eval Health Prof. 2017:163278717742189. 10.1177/0163278717742189 PMC6403020 29172702

[95] Hou F, Schimmele C. Stic k M. Statistics Canada. Changing demographics of racialized people in Canada. 2023. https://www150.statcan.gc.ca/n1/pub/36-28-0001/2023008/article/00001-eng.htm. Accessed 08 Jul 2025.

[96] Statista. Percentage distribution of income of full-time workers in Canada in 2022, by level of income. 2022. https://www.statista.com/statistics/464262/percentage-distribution-of-earnings-in-canada-by-level-of-income/#:~:text=In%202022%2C%2022.6%20percent%20of,100%2C000%20Canadian%20dollars%20or%20more. Accessed 08 Jul 2025.

